# Neurocognitive impairment in females with breast cancer treated with endocrine therapy and CDK4/6 inhibitors: a pharmacovigilance study using the World Health Organization’s database

**DOI:** 10.3389/fphar.2023.1278682

**Published:** 2023-10-20

**Authors:** Rachel Prevost, Basile Chretien, Elise-Marie Minoc, Charles Dolladille, Angélique Da-Silva, Ahmad Nehme, Florence Joly, Véronique Lelong-Boulouard, Etienne Bastien

**Affiliations:** ^1^ Department of Pharmacology, University Teaching Hospital of Caen-Normandie, Caen, France; ^2^ Normandie University, UNICAEN, INSERM COMETE, U1075, Caen, France; ^3^ Normandie University, UNICAEN, INSERM U1086 “Interdisciplinary Research Unit for Cancers Prevention and Treatment” (ANTICIPE), Caen, France; ^4^ Department of Neurology, University Teaching Hospital of Caen-Normandie, Caen, France; ^5^ Comprehensive Cancer Center Baclesse, Unicancer, Caen, France

**Keywords:** breast cancer, endocrine therapy, cyclin-dependent kinase 4/6 inhibitor, neurocognitive impairment, pharmacoepidemiology

## Abstract

**Importance:** Endocrine therapies (ETs) and inhibitors of cyclin-dependent kinases-4/6 (iCDK4/6s) are a standard treatment in breast cancer. However, data on potential neurocognitive impacts remain inconsistent for ET and are scarce for iCDK4/6s.

**Objective:** To evaluate whether ET and iCDK4/6s are associated with neurocognitive impairment (NCI).

**Methods:** We used observational, real-world cases of NCI from the World Health Organization’s database VigiBase^®^ to perform disproportionality analysis. Cases were defined as any symptom of NCI in females treated with ETs or iCDK4/6s. The study period was from the date of the first adverse event reported in VigiBase^®^ with iCDK4/6s (1 January 2014) until the date of data extraction (16 March 2022). In our primary analysis, we calculated the reporting odds ratio (ROR) adjusted for age to identify a potential association between NCI and individual ETs in isolation or in combination with iCDK4/6s. We also performed subgroup analyses by the NCI class.

**Results:** We identified 2.582 and 1.943 reports of NCI associated with ETs and iCDK4/6s, respectively. NCI was significantly associated with each ET [anastrozole: *n* = 405, aROR = 1.52 (95% CI: 1.37–1.67); letrozole: *n* = 741, aROR = 1.37 (95% CI: 1.27–1.47); exemestane: *n* = 316, aROR = 1.37 (95% CI: 1.22–1.53); tamoxifen: *n* = 311, aROR = 1.25 (95% CI: 1.12–1.40); and fulvestrant: *n* = 319, aROR = 1.19 (95% CI: 1.06–1.33)] and only with palbociclib for iCDK4/6s [*n* = 1,542, aROR = 1.41 (95% CI: 1.34–1.48)].

**Conclusion:** These findings suggest that in females treated for breast cancer, all ETs may be associated with NCI. However, amongst iCDK4/6s, NCI may be specific to palbociclib. NCI most frequently involved learning and memory as well as language. Neurocognitive impact of treatments requires better consideration and management.

## 1 Introduction

Endocrine therapies (ETs) have contributed to a significant increase in survival for females with breast cancer. Aromatase inhibitors (AIs) (anastrozole, letrozole, and exemestane), selective estrogen-receptor modulators and degraders (SERMs and SERDs) (tamoxifen, toremifene, and fulvestrant), and gonadotrophin-releasing hormone (GnRH) analogs (leuprorelin, goserelin, and triptorelin) are used in early or metastatic ER-positive breast cancer (Cardoso et al., 2019).

Inhibitors of cyclin-dependent kinases-4/6 (iCDK4/6s) have recently revolutionized the adjuvant and first line treatment of high-risk and metastatic ER-positive breast cancer. They are used in combination to improve the efficacy of ET by acting on the cell cycle checkpoint (Roskoski, 2016). The U.S. Food and Drug Administration (FDA) and the European Medicines Agency (EMA) have currently approved three iCDK4/6s: abemaciclib, palbociclib, and ribociclib (Ibrance, 2018; Kisqali, 2018; Verzenios, 2018; FDA, 2019a; FDA, 2022; FDA, 2023).

By means of all these therapeutic advances, patients survive longer and are treated for more extended periods of time. Therefore, they are potentially at risk for long-term adverse events (AEs). This raises questions regarding the impact of ET on quality of life and related outcomes (Franzoi et al., 2021; Haggstrom et al., 2022; Siegel et al., 2022). Numerous studies report neurocognitive impairment (NCI) with ET (Hugo and Ganguli, 2014; Haggstrom et al., 2022). However, the literature remains scarce and conclusions inconsistent (Lange et al., 2019; Haggstrom et al., 2022). Limited data are available regarding the impact of iCDK4/6s on cognition. Nevertheless, a recent review suggests that iCDK4/6s may negatively impact cognition (Kjoe et al., 2022).

Using neurocognitive symptoms reported in the World Health Organization’s (WHO) pharmacovigilance database VigiBase^®^, we performed a disproportionality analysis to evaluate the association between NCI and ETs in isolation or in combination with iCDK4/6s. In secondary analyses, we described the clinical features of NCI cases reported with ETs and iCDK4/6s.

## 2 Methods

### 2.1 Pharmacovigilance study procedure

We performed a pharmacovigilance study within VigiBase^®^, the largest pharmacovigilance database with more than 30 million reports received from more than 160 member countries. Vigibase^®^ has been developed to detect potential associations between drugs (including cancer treatments) and AEs (Guerrero et al., 2019; Briggs et al., 2022). AEs can be reported by healthcare or non-healthcare professionals, such as patients or manufacturers. Drugs are coded with the anatomical therapeutic chemical and AEs with the Medical Dictionary for Regulatory Activities (MedDRA). Cases were included when the imputability of NCI symptoms to ET and iCDK4/6s was defined as suspect/interacting/concomitant, using the WHODrug Global dictionary. Serious AE is defined as results in death, life threatening, require inpatient hospitalization or prolongation of existing hospitalization, results in persistent or significant disability, or at the judgment of the reporter.

As previously published, our query used the standardized MedDRA query and high-level group terms related to NCI: “dementia,” “mental impairment disorders,” “cognitive and attention disorders and disturbances,” “deliria,” “dementia and amnestic condition,” and “disturbances in thinking and perception” (Briggs et al., 2022; Gouverneur et al., 2023). In the absence of specific terms that describe drug-induced NCI and in order to avoid inclusion of neurological or psychiatric diseases, we focused our query on symptoms (Supplementary Tables S1A) and excluded all preferred terms (PTs) related to neurological or psychiatric diseases (Supplementary Tables S1B). We also classified (EMM and VLB) each of the PTs included in our study into one of the six NCI patterns defined by the Diagnostic and Statistical Manual of Mental Disorders, Fifth Edition (DSM-5): social cognition, language, executive function, complex attention, learning and memory, and perceptual motor function (Sachdev et al., 2014).

The analysis included ETs (“letrozole,” “anastrozole,” “exemestane,” “tamoxifen,” “toremifene,” and “fulvestrant”) and iCDK4/6s (“palbociclib,” “abemaciclib,” and “ribociclib”). One drug may be associated with several PTs. We excluded GnRH analogs due to their multiple non-oncological indications. To minimize the risk of non-breast cancer indications of ETs and iCDK4/6s, the study population was restricted to females.

The protocol was approved by a hospital committee with competency for research not requiring authorization by an institutional review board (University of Caen Normandy, France; reference: 2646, dated 15 July 2021).

### 2.2 Statistical analysis and outcomes

We performed a pharmacovigilance disproportionality analysis using R version 4.2.1. Disproportionality analysis is performed to compare the proportion of reporting of a specific AE with a drug of interest to the expected proportion assuming the AE with this drug of interest and is independently reported (Faillie, 2019).

In our primary analysis, we first calculated the reporting odds ratio (ROR) adjusted for age to identify a potential association between NCI and each ET. Second, we calculated ROR adjusted for age to identify a potential association between NCI and each iCDK4/6. In the primary analysis, we restricted the period for the extraction of cases and non-cases from the date of the first AE reported in VigiBase^®^ with iCDK4/6s (1 January 2014) until the date of data extraction (16 March 2022). Case characteristics were summarized with means for quantitative variables and proportions for qualitative variables. First, to avoid confounding by the presence of breast cancer in cases, we performed a sensibility analysis where we restricted non-cases to reports that include antineoplastic agents, ET and immunotherapy. Second, to avoid signal induced by reporters other than health care professionals, we repeated the analyses after restricting reports to those from health care professionals. Third, for cases exposed to ETs, to avoid confounding by the co-prescription of an iCDK4/6, we performed an additional analysis where we extracted cases and non-cases from the date of the first AE reported for each ET to the date of the first AE reported with an iCDK4/6. Fourth, a sensibility analysis was also performed after excluding reports with co-illnesses and co-treatments known to cause NCI (Supplementary Table 2).

For the primary and secondary analyses, we report 95% credibility intervals (CIs), with a lower ROR CI bound >1 denoting an association between a drug and an AE. For the effective interpretation of the signal, RORs were only calculated if there were at least five reports for a drug of interest/AE pair.

## 3 Results

**FIGURE 1:**
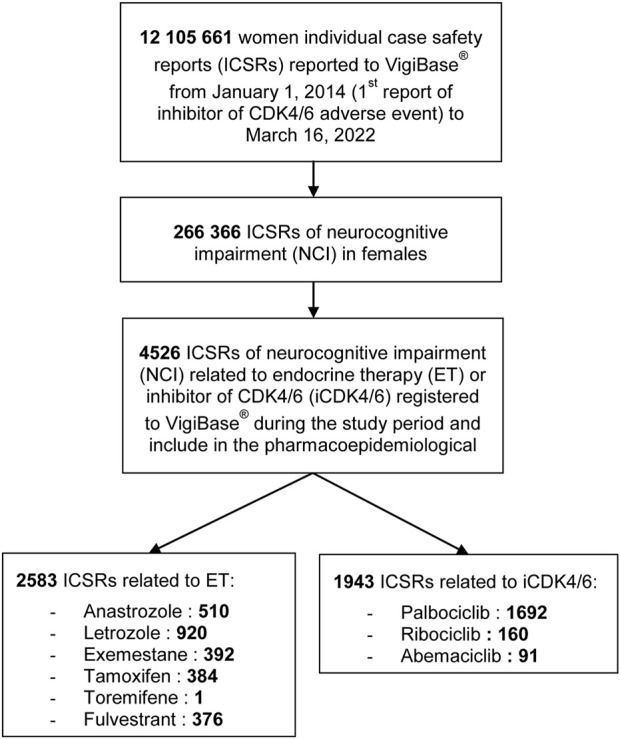
Flow chart of the pharmacovigilance study.

During the study period (1 January 2014 to 16 March 2022), a total of 12,105,661 AEs were reported in Vigibase^®^. We identified 262,366 reports related to NCI, of which 2,583 concerned ETs and 1,943 concerned iCDK4/6s (Figure 1). A total of 3,400 reports came from the Americas (75%), 971 from Europe (21%), 64 from Eastern Mediterranean (1%), 52 from Asia (1%), and 39 from Africa (<1%). The most reported PTs were “memory impairment” (27%), “amnesia” (9%), “cognitive disorder” (6%), “disturbance in attention” (6%), and “speech disorder (5%) (Supplementary Tables S1A). After excluding cases without age information, we included 2,093 reports concerning ETs and 1,686 reports concerning iCDK4/6s in the age-adjusted primary analysis.

Reports of NCI in patients treated with ET and/or iCDK4/6s concerned all age classes: 23% of females were more than 75 years old (*n* = 800), 26% were between 65 and 74 years old (*n* = 884), 29% were between 45 and 64 years old (*n* = 1,009), and 5% were between 18 and 44 years old (*n* = 158). Age was missing in 17% of reports (*n* = 595). According to the WHO classification, 57% of reports related to NCI were considered serious by reporters. Treatment was interrupted in one-third of patients with serious NCI. Data on time to onset, reversibility after interruption, and treatment rechallenges were not available.

Regarding ET, anastrozole (aROR 1.52; 95% CI: 1.37–1.67), letrozole (aROR 1.37; 95% CI: 1.27–1.47), exemestane (aROR 1.37; 95% CI: 1.22–1.53), tamoxifen (aROR 1.25; 95% CI: 1.12–1.40), and fulvestrant (aROR 1.19; 95% CI: 1.06–1.33) were significantly associated with higher reporting of NCI (Table 1). Only 207 reports were available for toremifene and one included NCI, so no disproportionality was performed.

**TABLE 1:** Disproportionality analysis of neurocognitive impairment with individual endocrine therapies and inhibitors of CDK4/6.

	Drug	N_observed_	N_drug_	ROR	95% CI
AIs	Anastrozole	405	11,729	1.52	(**1.37**–1.67)
	Letrozole	741	23,957	1.37	(**1.27**–1.47)
	Exemestane	316	10,152	1.37	(**1.22**–1.53)
SERM	Tamoxifen	311	11,941	1.25	(**1.12**–1.40)
SERD	Fulvestrant	319	11,706	1.19	(**1.06**–1.33)
iCDK4/6s	Palbociclib	1,542	47,424	1.41	(**1.34**–1.48)
	Ribociclib	81	5,064	0.73	(0.59–0.91)
	Abemaciclib	63	4,343	0.65	(0.51–0.83)

The reporting odds ratio (ROR, adjusted for age) and its 95% credibility interval (CI) lower and upper endpoints evaluate the observed-to-expected ratios of neurocognitive impairment (NCI) cases associated with endocrine therapies and inhibitors of CDK4/6 in VigiBase^®^ (from 1 January 2014 to 16 March 2022). A lower ROR CI endpoint >1 (**in bold**) denotes an association between a drug and an adverse event. Only one case of NCI was identified with toremifene. ROR was not calculable and is therefore not presented in the table.

AIs, aromatase inhibitors; N_drug_, number of AEs with the drug over the period of interest; N_observed_, number of NCI events with the drug over the period of interest; SERM, selective estrogen-receptor modulator; SERD, selective estrogen-receptor degrader.

Regarding iCDK4/6s, only palbociclib (aROR 1.41; 95% CI: 1.34–1.48) was significantly associated with a higher reporting of NCI. No signal was found for ribociclib (aROR 0.73; 95% CI: 0.59–0.91) and abemaciclib (aROR 0.65; 95% CI: 0.51–0.83).

After restricting non-cases to reports that include antineoplastic agents, ET, and immunotherapy, results were broadly consistent with the primary analysis except for tamoxifen (ROR 1.07; 95% CI: 0.96–1.18) and fulvestrant (ROR 1.08; 95% CI: 0.97–1.19), which were no longer statistically significant (Supplementary Table S3). After restricting reports to those from healthcare professionals, the results were broadly consistent with the primary analysis except for tamoxifen (ROR 1.11; 95% CI: 0.96–1.28), exemestane (ROR 1.09; 95% CI: 0.94–1.26), and fulvestrant (ROR 1.07; 95% CI: 0.92–1.23), which were no longer statistically significant (Supplementary Table S4). In the additional analysis that only included reports made prior to the first use of iCDK4/6s, the results were no longer statistically significant for letrozole (ROR 1.10; 95% CI: 0.99–1.23), exemestane (ROR 1.01; 95% CI: 0.86–1.18), and tamoxifen (ROR 0.91; 95% CI: 0.84–0.99) (Supplementary Table S5). Co-illnesses and co-treatments known to be associated with NCI were, respectively, present in 84 and 31 of the 4,524 reports. Due to their low number, sensitivity analyses excluding co-illnesses and co-treatments were not performed.

To better describe the cases of NCI with ETs and iCDK4/6s identified in the primary analysis, we calculated aROR for each of the six NCI patterns (Table 2). Anastrozole was significantly associated with a higher reporting of complex attention (aROR 1.36; 95% CI: 1.15–1.60), language (aROR 1.95; 95% CI: 1.65–2.30), and perceptual motor function impairments (aROR 1.25; 95% CI: 1.03–1.52). Letrozole and exemestane were associated with a higher reporting of language (aROR 2.11; 95% CI: 1.89–2.36 and aROR 2.18; 95% CI: 1.84–2.57, respectively) and learning and memory impairments (aROR 1.54; 95% CI: 1.22–1.94 and aROR 1.78; 95% CI: 1.28–2.48, respectively). Tamoxifen and fulvestrant were only associated with a higher reporting of language impairment (aROR 1.39; 95% CI: 1.14–1.70 and aROR 1.92; 95% CI: 1.63–2.27, respectively). Palbociclib was significantly associated with a higher reporting of language and learning and memory impairments (aROR 2.74; 95% CI: 2.55–2.94 and aROR 1.31; 95% CI: 1.10–1.57, respectively). No drugs were associated with a higher reporting of executive function or social cognition impairment.

**TABLE 2:** Analysis of neurocognitive impairment classes with individual endocrine therapies and inhibitors of CDK4/6.

Neurocognitive impairment subtype	Drug	N_observed_	N_drug_	aROR	95% CI
Complex attention	Anastrozole	145	11,989	1.36	(**1.15**–1.60)
	Letrozole	238	24,460	1.10	(0.97–1.25)
	Exemestane	99	10,369	1.07	(0.88–1.31)
	Tamoxifen	115	12,137	1.20	(0.99–1.44)
	Fulvestrant	105	11,920	0.98	(0.81–1.18)
	Palbociclib	415	48,551	0.94	(0.85–1.03)
	Ribociclib	20	5,125	0.46	(0.30–0.72)
	Abemaciclib	23	4,383	0.61	(0.40–0.91)
Executive function	Anastrozole	8	12,126	1.06	(0.53–2.12)
	Letrozole	21	24,677	1.36	(0.88–2.08)
	Exemestane	6	10,462	0.92	(0.41–2.05)
	Tamoxifen	12	12,240	1.46	(0.83–2.57)
	Fulvestrant	8	12,017	1.07	(0.54–2.15)
	Palbociclib	26	48,940	0.85	(0.58–1.26)
	Ribociclib	4	5,141	NC	NC
	Abemaciclib	4	4,402	NC	NC
Language	Anastrozole	26	12,108	1.95	(**1.65**–2.30)
	Letrozole	71	24,627	2.11	(**1.89**–2.36)
	Exemestane	35	10,433	2.18	(**1.84**–2.57)
	Tamoxifen	27	12,225	1.39	(**1.14**–1.70)
	Fulvestrant	26	11,999	1.92	(**1.63**–2.27)
	Palbociclib	122	48,844	2.74	(**2.55**–2.94)
	Ribociclib	9	5,136	1.10	(0.79–1.55)
	Abemaciclib	3	4,403	NC	NC
Learning and memory	Anastrozole	145	11,989	1.13	(0.77–1.67)
	Letrozole	315	24,383	1.54	(**1.22**–1.94)
	Exemestane	139	10,329	1.78	(**1.28**–2.48)
	Tamoxifen	99	12,153	1.27	(0.87–1.85)
	Fulvestrant	141	11,884	1.14	(0.78–1.68)
	Palbociclib	805	48,161	1.31	(**1.10**–1.57)
	Ribociclib	34	5,111	0.97	(0.50–1.86)
	Abemaciclib	18	4,388	0.37	(0.12–1.14)
Perceptual motor function	Anastrozole	101	12,033	1.25	(**1.03**–1.52)
	Letrozole	115	24,583	0.70	(0.58–0.84)
	Exemestane	50	10,418	0.71	(0.54–0.94)
	Tamoxifen	69	12,183	0.98	(0.77–1.24)
	Fulvestrant	48	11,977	0.58	(0.44–0.78)
	Palbociclib	120	48,846	0.35	(0.29–0.42)
	Ribociclib	9	5,136	0.28	(0.15–0.54)
	Abemaciclib	16	4,390	0.56	(0.34–0.92)
Social cognition	Anastrozole	0	12,134	NC	NC
	Letrozole	1	24,697	NC	NC
	Exemestane	1	10,467	NC	NC
	Tamoxifen	0	12,252	NC	NC
	Fulvestrant	1	12,024	NC	NC
	Palbociclib	1	48,965	NC	NC
	Ribociclib	0	5,145	NC	NC
	Abemaciclib	0	4,406	NC	NC

The reporting odds ratio (ROR, adjusted for age) and its 95% credibility interval (CI) lower and upper endpoints evaluate the observed-to-expected ratios of neurocognitive impairment (NCI) cases associated with endocrine therapies and inhibitors of CDK4/6 in VigiBase^®^ (from 1 January 2014 to 16 March 2022). A lower ROR CI endpoint >1 (**in bold**) denotes an association between a drug and an adverse event. Only one case of NCI was identified with toremifene. ROR was not calculable and is therefore not presented in the table.

AIs, aromatase inhibitors; N_drug_, number of AEs with the drug over the period of interest; NC, not calculable (when the number of reports was less than five); N_observed_, number of NCI events with the drug over the period of interest; SERM, selective estrogen-receptor modulator; SERD, selective estrogen-receptor degrader.

## 4 Discussion

Using pharmacovigilance data, we identified a significant association between reporting of NCI and AIs, tamoxifen, fulvestrant, and palbociclib. No signal was observed with the other iCDK4/6s. NCI was mainly related to learning and memory as well as language. Reports were not limited to the elderly as one-third concerned females under the age of 65.

### 4.1 Endocrine therapies

These results provide further evidence that all ET classes may be associated with NCI in females with breast cancer. Our results for AIs are consistent with a meta-analysis of studies that used neurocognitive tests and found the association between AIs and verbal and learning/memory impairments in females with breast cancer (Underwood et al., 2018). Our results suggest that anastrozole and exemestane could also be associated with language impairment. Several studies that used neurocognitive tests support that tamoxifen may negatively impact cognition and language and attention impairments, which is consistent with the findings of our study (Schilder et al., 2010; Boele et al., 2015). Based on neuropsychological assessments of females in randomized clinical trials that compared tamoxifen to AIs, several studies suggest that tamoxifen may lead to NCI more than AIs. In the TEAM trial, females treated with adjuvant tamoxifen had slower information processing speed than those treated with exemestane (*p* = 0.02; Cohen’s d = 0.36) (Schilder et al., 2010). Similarly, overall cognition of females treated with adjuvant tamoxifen in the BIG-1-98 trial was worse than those treated with letrozole (*p* = 0.04, Cohen’s d = 0.40) (Phillips et al., 2010). However, due to discordant results, the impact of AIs and tamoxifen on cognition remains controversial (Lange et al., 2019; Novick et al., 2020). Due to the absence of prior clinical studies aiming to assess the neurocognitive impact of fulvestrant therapy, our study provides relevant data on its neurocognitive consequences (Robertson et al., 2016).

GnRH agonists were not investigated in this study due to their wider range of indications. Data regarding the neurocognitive effect of leuprorelin in females with breast cancer remain scarce. Meta-analyses evaluated the effect of leuprorelin on cognition in men with prostate cancer and showed discordant results (Nead et al., 2017; Sun et al., 2018). Using a similar pharmacovigilance methodology, a recent study highlighted an association between androgen deprivation therapies and NCI in prostate cancer, including leuprorelin (ROR 1.47, 95% CI: 1.34; 1.62) (Briggs et al., 2022).

Estrogen receptors are expressed both on breast cancer cells and the central nervous system. Estradiol is involved in central neurotransmission and could have an impact on axonal growth. ETs may lead to NCI due to decreased estradiol activation in areas involved in cognition functions, such as the hypothalamus, amygdala, or hippocampus (Lange et al., 2019; Haggstrom et al., 2022).

### 4.2 Inhibitors of cyclin-dependent kinases-4/6

Concerning iCDK4/6s, our study identified a signal of NCI associated with palbociclib, mainly related with language and learning and memory impairments. In the PEARL trial, the cognition subscale of the European Organization for the Research and Treatment of Cancer Quality of Life Questionnaire (EORTC-QLQ-C30) favored the palbociclib plus ET arm (hazard ratio = 0.70; 95% CI: 0.54–0.89) (Kahan et al., 2021). However the control arm was capecitabine, which may itself lead to NCI (Lange et al., 2019). In the PALOMA-3 trial, there was no difference in cognitive outcomes between the palbociclib and fulvestrant arms compared to the placebo and fulvestrant arms (Harbeck et al., 2021). However, the EORTC-QLQ-C30 cognition subscale is only based on two out of thirty questions and concerns self-reported cognitive impairment. The absence of a signal with abemaciclib is concordant with the results of the MONARCH-2 trial (Kaufman et al., 2020). For ribociclib, the EORTC-QLQ-C30 cognition subscale was not published in MONALEESA-3 trial. Finally, abemaciclib and ribociclib are reported more recently, and the number of reports is lower. However, the design of disproportionality analyses enables to detect signals with a low number of AEs (Cellier et al., 2023). Moreover, there was no trend toward significance for abemaciclib and ribociclib that might suggest a lack of power.

Whether iCDK4/6s affect the central nervous system is unknown. Cyclin D inhibition may alter neurogenesis and lead to NCI (Urbach and Witte, 2019; Kjoe et al., 2022). However, this does not explain why the signal in our study was isolated to palbociclib. Considering that abemaciclib has good penetrance into the central nervous system, pharmacokinetic parameters likely do not explain this potential differential effect (Hendrychová et al., 2021). The differential kinase affinity spectrum could explain why NCI may be specific to palbociclib. In contrast to abemaciclib and ribociclib, palbociclib inhibits the tropomyosin receptor kinase (TRK), encoded by the *neurotrophic receptor tyrosine kinase* (*NTRK*) genes (Hendrychová et al., 2021). Binding to the brain-derived neurotrophic factor (BDNF), TRK is a tyrosine kinase receptor which regulates neuronal development, differentiation, and survival, including those in the hippocampus. In *in vitro*, BDNF/TRK deprivation is associated with elevations of the β-amyloid peptide in hippocampal neurons, leading to apoptotic death (Matrone et al., 2008). In mice, deficiency of this pathway is associated with neuro-inflammation and impairment of memory and learning (Wang et al., 2019). In humans, reduced BDNF mRNA expression has been observed during post-mortem examination of patients with Alzheimer’s disease (Connor et al., 1997). NTRK fusions are observed in 0.3% of lung cancers and are targeted by two TRK inhibitors approved by the FDA: entrectinib and larotrectinib (Harada et al., 2021). Based on clinical trials, SmPCs of entrectinib and larotrectinib report NCI in, respectively, 27% and 1% of patients. Further studies are needed to determine whether inhibition of the BDNF/TRK pathway mediates NCI in patients treated with palbociclib (FDA, 2019b; FDA, 2019c).

### 4.3 Strengths and limitations

The present study investigated the association between NCI and ET in isolation or in combination with iCDK4/6s in females with breast cancer using the worldwide pharmacovigilance database Vigibase^®^, allowing us to isolate 4,526 reports of NCI associated with ET and/or iCDK4/6s. Our primary analysis was adjusted for age and is strengthened by the sensitivity analyses, suggesting that our results are not driven by age or the presence of breast cancer. Sensitivity analysis regarding the type of the reporter was less consistent, probably due to lack of power. Our secondary analysis using DSM-5 neurocognitive patterns allowed a finer description of the NCI symptoms associated with ET and palbociclib. Moreover, we used an original and complementary approach based on pharmacovigilance reports of neurocognitive symptoms rather than neuropsychological tests or self-questionnaires, such as EORTC-QLQ-C30, which are used in clinical trials (Underwood et al., 2018; Kaufman et al., 2020; Harbeck et al., 2021).

Our study had several limitations. Inherent to pharmacovigilance databases is missing data, which did not allow us to more comprehensively describe the reports. Moreover, we were unable to determine the line of treatment, previous chemotherapy, and co-prescribed ET with iCDK4/6, which could have biased the signal. In addition, Vigibase^®^ does not allow access to medical records to confirm the diagnosis of NCI and eliminate differential diagnosis (such as depression and cerebral metastases). To limit non-breast cancer indications, we restricted our analysis to cases in females. It would be necessary to conduct similar analyses to extend these results to males. Last, disproportionality pharmacovigilance analyses identify new AE signals that require confirmation with the high level of evidence studies (Faillie, 2019). They are also necessary to determine the incidence of NCI.

## 5 Conclusion

This pharmacovigilance study strengthens the association between ET and NCI in females with breast cancer. We highlighted a new signal for iCDK4/6s isolated to palbociclib, which requires further research. NCI impacted all exposed age groups and mostly involved learning, memory, and language. Neurocognitive impact of breast cancer treatments must be better considered. NCI management involves non-pharmacological approaches that need to be developed.

## Data Availability

Publicly available datasets were analyzed in this study. These data can be found at: public access to overview statistics from VigiBase® can be gained through the VigiAccess website, http://www.vigiaccess.org/.
