# Supplementary material for: Neurocognitive impairment in females with breast cancer treated with endocrine therapy and CDK4/6 inhibitors: a pharmacovigilance study using the World Health Organization’s database
Source: Front Pharmacol. 2023 Oct 20;14:1278682. doi: 10.3389/fphar.2023.1278682 (PMC10622981; doi:10.3389/fphar.2023.1278682)
Supplement: Supplementary file 1 [file DataSheet1.docx]

Supplementary Material

# Supplementary Tables

**Supplementary table 1: List of Preferred Terms from the Standard MedDRA Query “Dementia” and High Level Group Terms “mental impairments disorders, “cognitive and attention disorders and disturbance”, “deliria”, “dementia and amnestic condition”, “disturbance”.**

1. **List of preferred terms included in the Vigibase® query to identify neurocognitive impairment reports. Preferred terms were matched to DSM-5 neurocognitive patterns.** Only preferred terms corresponding to symptoms were including the Vigibase® query.

| Preferred terms | Neurocognitive pattern | Number of occurrences |
| --- | --- | --- |
| Disturbance in attention | Complex attention | 281 |
| Delirium | Complex attention | 31 |
| Attention deficit hyperactivity disorder | Complex attention | 22 |
| Bradyphrenia | Complex attention | 15 |
| Distractibility | Complex attention | 11 |
| Tachyphrenia | Complex attention | 6 |
| Atypical attention deficit syndrome | Complex attention | 0 |
| Thought broadcasting | Complex attention | 0 |
| Flight of ideas | Complex attention | 0 |
| Change in sustained attention | Complex attention | 0 |
| Confusional state | Complex attention | 0 |
| Apathy | Executive function | 58 |
| Mental fatigue | Executive function | 17 |
| Reading disorder | Executive function | 16 |
| Thought blocking | Executive function | 3 |
| Abulia | Executive function | 2 |
| Impaired reasoning | Executive function | 1 |
| Transient aphasia | Executive function | 0 |
| Perseveration | Executive function | 0 |
| Visual agnosia | Executive function | 0 |
| Executive dysfunction | Executive function | 0 |
| Speech disorder | Language | 227 |
| Incoherent | Language | 14 |
| Cognitive linguistic deficit | Language | 0 |
| Circumstantiality | Language | 0 |
| Derailment | Language | 0 |
| Poverty of thought content | Language | 0 |
| Memory impairment | Learning and memory | 1230 |
| Amnesia | Learning and memory | 435 |
| Amnestic disorder | Learning and memory | 4 |
| Learning disability | Learning and memory | 4 |
| Dyslexia | Learning and memory | 3 |
| Learning disorder | Learning and memory | 3 |
| Confabulation | Learning and memory | 2 |
| Transient global amnesia | Learning and memory | 2 |
| Paramnesia | Learning and memory | 1 |
| Alexia | Learning and memory | 1 |
| Anterograde amnesia | Learning and memory | 1 |
| Acalculia | Learning and memory | 0 |
| Thought withdrawal | Learning and memory | 0 |
| Loose associations | Learning and memory | 0 |
| Retrograde amnesia | Learning and memory | 0 |
| Hallucination | Perceptual-motor function | 93 |
| Disorientation | Perceptual-motor function | 86 |
| Formication | Perceptual-motor function | 45 |
| Psychomotor hyperactivity | Perceptual-motor function | 26 |
| Delusion | Perceptual-motor function | 20 |
| Hallucination, visual | Perceptual-motor function | 14 |
| Hallucination, auditory | Perceptual-motor function | 13 |
| Daydreaming | Perceptual-motor function | 11 |
| Near death experience | Perceptual-motor function | 8 |
| Illusion | Perceptual-motor function | 5 |
| Derealisation | Perceptual-motor function | 3 |
| Prosopagnosia | Perceptual-motor function | 2 |
| Persecutory delusion | Perceptual-motor function | 2 |
| Hallucination, olfactory | Perceptual-motor function | 2 |
| Delusional perception | Perceptual-motor function | 2 |
| Apraxia | Perceptual-motor function | 2 |
| Delusional disorder, unspecified type | Perceptual-motor function | 2 |
| Psychomotor disadaptation syndrome | Perceptual-motor function | 1 |
| Agraphia | Perceptual-motor function | 1 |
| Amusia | Perceptual-motor function | 1 |
| Hallucination, tactile | Perceptual-motor function | 1 |
| Somatic hallucination | Perceptual-motor function | 1 |
| Deja vu | Perceptual-motor function | 1 |
| Flashback | Perceptual-motor function | 1 |
| Autoscopy | Perceptual-motor function | 1 |
| Time perception altered | Perceptual-motor function | 1 |
| Hemiapraxia | Perceptual-motor function | 0 |
| Dyscalculia | Perceptual-motor function | 0 |
| Anosognosia | Perceptual-motor function | 0 |
| Altered pitch perception | Perceptual-motor function | 0 |
| Hemiasomatognosia | Perceptual-motor function | 0 |
| Delusion of grandeur | Perceptual-motor function | 0 |
| Delusion of parasitosis | Perceptual-motor function | 0 |
| Delusion of reference | Perceptual-motor function | 0 |
| Delusion of replacement | Perceptual-motor function | 0 |
| Erotomanic delusion | Perceptual-motor function | 0 |
| Jealous delusion | Perceptual-motor function | 0 |
| Somatic delusion | Perceptual-motor function | 0 |
| Thought insertion | Perceptual-motor function | 0 |
| Depressive delusion | Perceptual-motor function | 0 |
| Mixed delusion | Perceptual-motor function | 0 |
| Hallucination, gustatory | Perceptual-motor function | 0 |
| Hallucination, synaesthetic | Perceptual-motor function | 0 |
| Paroxysmal perceptual alteration | Perceptual-motor function | 0 |
| Imperception | Perceptual-motor function | 0 |
| Jamais vu | Perceptual-motor function | 0 |
| Hallucinations, mixed | Perceptual-motor function | 0 |
| Pseudohallucination | Perceptual-motor function | 0 |
| Aberrant motor behaviour | Perceptual-motor function | 0 |
| Agnosia | Perceptual-motor function | 0 |
| Delusion of theft | Perceptual-motor function | 0 |
| Visuospatial deficit | Perceptual-motor function | 0 |
| Judgement impaired | Social cognition | 4 |
| Inappropriate affect | Social cognition | 4 |
| Disturbance in social behaviour | Social cognition | 1 |
| Illogical thinking | Social cognition | 0 |
| Magical thinking | Social cognition | 0 |
| Tangentiality | Social cognition | 0 |
| Paralogism | Social cognition | 0 |
| Cognitive disorder | Unclassified | 309 |
| Mental disorder | Unclassified | 163 |
| Mental impairment | Unclassified | 111 |

1. **List of excluded preferred terms from the Vigibase® query:**

- Preferred terms from broad spectrum of the standard MeDRA query “Dementia” and non-symptoms preferred terms were excluded from Vigibase® query.

| Corticobasal degeneration | Familial scaphocephaly syndrome | Morbid thoughts |
| --- | --- | --- |
| Korsakoff's syndrome | Johanson-Blizzard syndrome | Intellectualisation |
| Vascular dementia | Cornelia de Lange syndrome | Pathological doubt |
| Vascular cognitive impairment | Smith-Lemli-Opitz syndrome | Intrusive thoughts |
| Posterior cortical atrophy | Allan-Herndon-Dudley syndrome | Charles Bonnet syndrome |
| Wernicke-Korsakoff syndrome | 2-Hydroxyglutaric aciduria | Phantom vibration syndrome |
| Creutzfeldt-Jakob disease | Emanuel syndrome | Restlessness |
| Dementia with Lewy bodies | Kleefstra syndrome | Thinking abnormal |
| Early onset familial Alzheimer's disease | Trisomy 4p | Hippocampal sclerosis |
| Prion disease | Pallister-Killian syndrome | Agitation |
| Progressive supranuclear palsy | DOOR syndrome | Mental status changes |
| Scatolia | Zhu-Tokita-Takenouchi-Kim syndrome | Neuropsychiatric symptoms |
| Variant Creutzfeldt-Jakob disease | Gatad2b associated neurodevelopmental disorder | Cerebellar cognitive affective syndrome |
| Initial insomnia | Benjamin syndrome | Right hemisphere deficit syndrome |
| Neuritic plaques | Griscelli syndrome | Hippocampal atrophy |
| Patient elopement | ACAD9 deficiency | Mixed dementia |
| Somnambulism | Smith-Magenis syndrome | Cerebral atrophy |
| Alcohol induced persisting dementia | Coffin-Lowry syndrome | Neuropsychological test abnormal |
| HIV-associated neurocognitive disorder | Shprintzen-Goldberg syndrome | Personality change |
| Membranous lipodystrophy | Alpha-thalassaemia-intellectual deficit syndrome | Dementia of the Alzheimer's type, with delirium |
| Brunner syndrome | X-linked intellectual disability, Siderius type | Dementia of the Alzheimer's type, with delusions |
| Infantile spasms | SADDAN syndrome | Pseudodementia |
| Lennox-Gastaut syndrome | 1p36 deletion syndrome | Prodromal Alzheimer's disease |
| Benign rolandic epilepsy | Cardiofaciocutaneous syndrome | Dementia |
| Early infantile epileptic encephalopathy with burst-suppression | Bosch-Boonstra-Schaaf optic atrophy syndrome | Dementia Alzheimer's type |
| CDKL5 deficiency disorder | SATB2-associated syndrome | Dementia of the Alzheimer's type, uncomplicated |
| Neurologic neglect syndrome | Weaver syndrome | Presenile dementia |
| Gerstmann's syndrome | Aniridia-cerebellar ataxia-mental deficiency | Senile dementia |
| Central auditory processing disorder | Ablepharon macrostomia syndrome | Mini mental status examination abnormal |
| Alien limb syndrome | Acrocallosal syndrome | Clinical dementia rating scale score abnormal |
| Simultanagnosia | Coffin Siris syndrome | Dementia of the Alzheimer's type, with depressed mood |
| Cri du Chat syndrome | Filippi syndrome | Oppositional defiant disorder |
| Fragile X syndrome | Fountain syndrome | Disruptive mood dysregulation disorder |
| Galactosaemia | De Barsy syndrome | Frontotemporal dementia |
| Phenylketonuria | Opitz trigonocephaly syndrome | Aphasia |
| Prader-Willi syndrome | Creatine deficiency syndrome | Borderline mental impairment |
| Rubinstein-Taybi syndrome | Pallister W syndrome | Abnormal behaviour |
| Trisomy 21 | Roberts syndrome | Affect lability |
| Trisomy 22 | Otopalatodigital spectrum disorder | Aggression |
| Sjogren-Larsson syndrome | KBG syndrome | Behaviour disorder |
| Cohen syndrome | DDX3X syndrome | Defiant behaviour |
| Williams syndrome | Zeichi-Ceide syndrome | Disinhibition |
| Wolf-Hirschhorn syndrome | Postictal state | Feeling abnormal |
| Langer-Giedion syndrome | Confusion postoperative | Flat affect |
| Oculocerebrorenal syndrome | Preictal state | Hostility |
| Osteoporosis-pseudoglioma syndrome | Immune effector cell-associated neurotoxicity syndrome | Hypomania |
| Greig's syndrome | Delirium tremens | Intelligence test abnormal |
| Trisomy 18 | Delirium febrile | Loss of personal independence in daily activities |
| Trisomy 17 | Post-injection delirium sedation syndrome | Mood altered |
| Laurence-Moon-Bardet-Biedl syndrome | Postoperative delirium | Mood swings |
| Dubowitz syndrome | Intensive care unit delirium | Morose |
| Schinzel-Giedion syndrome | Post-traumatic amnestic disorder | Negativism |
| Kabuki make-up syndrome | Transient epileptic amnesia | Primary progressive aphasia |
| CHARGE syndrome | Kuru | Psychotic behaviour |
| Velo-cardio-facial syndrome | Baltic myoclonic epilepsy | Psychotic disorder |
| Intellectual disability | Gerstmann Straussler Scheinker syndrome | Sexually inappropriate behaviour |
| Adiposogenital dystrophy | Primary familial brain calcification | Social avoidant behaviour |
| Tetrahydrobiopterin deficiency | CSF HIV escape syndrome | Somnolence |
| Norrie's disease | Sneddon's syndrome | Sopor |
| Trisomy 14 | Vascular encephalopathy | Suspiciousness |
| Mevalonic aciduria | Cotard's syndrome |  |
| Ideas of reference | Symbolic dysfunction |  |

**Supplementary table 2: List of co-illnesses and co-treatments defined *a priori* that are associated with neurocognitive impairment.**

1. **Predefined list of co-illnesses associated with neurocognitive impairment.**

Co-illnesses using preferred terms were defined beforehand by consensus of our team of geriatricians, neuropharmacologists and oncologists (EB, EMM, FJ & VLB). Co-illnesses were extracted from the co-treatment reported indications.

| Alcohol abuse | Hypoacusis |
| --- | --- |
| Brain neoplasm | Infection |
| Cerebrovascular accident | Lens disorders |
| Dementia Alzheimer’s type | Optic nerve disorders |
| Dementia with Lewy bodies | Parkinson’s disease |
| Drug abuse | Radiotherapy to brain |
| Epilepsy | Retinal disorder |
| Fecaloma | Urinary retention |
| Glaucoma | Vitamin B12 deficiency |
| Hypercalcemia |  |

1. **Predefined list of co-treatments associated with neurocognitive impairment.**

Co-treatments using Anatomical Therapeutic Chemical Classification System classes were defined beforehand by consensus of our team of geriatricians, neuropharmacologists and oncologists (EB, EMM, FJ & VLB). “Anticholinergic drugs” were subsequently added to this list and identified by consensus (EB, BC).

| Anticholinergic drugs |
| --- |
| Anti-Parkinson drugs |
| Corticosteroids for systemic use |
| Opioids |
| Psychoanaleptics |
| Psycholeptics |

**Supplementary table 3: Disproportionality analysis of neurocognitive impairments with individual endocrine therapies and inhibitors of CDK4/6 after restricting non-cases to antineoplastic agents, endocrine therapy and immunotherapy reports**

The relative odds ratio (ROR) and its 95% credibility interval (CI) lower and upper endpoints evaluate the observed-to-expected ratios of neurocognitive impairment (NCI) cases associated with ET and iCDK4/6 in VigiBase® (from January 1^st^, 2014 to March 16, 2022). A lower ROR CI endpoint >1 (**in bold** **type**) denotes an association between a drug and an adverse event. Only one case of NCI was identified with toremifene. A ROR was not calculable and is therefore not presented in the table. AI: aromatase inhibitors N_drug_: number of adverse events with the drug over the period of interest; N_observed_: number of reports of NCI with the drug over the period of interest; SERM: selective estrogen receptor modulator; SERD: selective estrogen receptor degrader.

|  | Drug | N_observed_ | N_drug_ | ROR | 95% CI |
| --- | --- | --- | --- | --- | --- |
| AIs | Anastrozole | 510 | 14825 | 1.52 | (**1.39**-1.66) |
|  | Letrozole | 920 | 31840 | 1.28 | (**1.20**-1.37) |
|  | Exemestane | 392 | 14117 | 1.22 | (**1.11**-1.35) |
| SERM | Tamoxifen | 384 | 15841 | 1.07 | (0.96-1.18) |
| SERD | Fulvestrant | 376 | 15393 | 1.08 | (0.97-1.19) |
| iCDK4/6 | Palbociclib | 1692 | 55761 | 1.35 | (**1.29**-1.42) |
|  | Ribociclib | 160 | 10376 | 0.68 | (0.58-0.79) |
|  | Abemaciclib | 91 | 6952 | 0.58 | (0.47-0.71) |

**Supplementary table 4: Disproportionality analysis of neurocognitive impairment with individual endocrine therapies and inhibitors of CDK4/6, restricting reports to those from health care professionals**

The relative odds ratio (ROR) and its 95% credibility interval (CI) lower and upper endpoints evaluate the observed-to-expected ratios of neurocognitive impairment (NCI) cases associated with endocrine therapies and inhibitors of CDK4/6 in VigiBase® (from January 1^st^, 2014 to March 16, 2022). A lower ROR CI endpoint >1 (**in bold** **type**) denotes an association between a drug and an adverse event. Only one case of NCI was identified with toremifene. A ROR was not calculable and is therefore not presented in the table. AI: aromatase inhibitors; N_drug_: number of adverse events with the drug over the period of interest; N_observed_: number of reports of NCI with the drug over the period of interest; SERM: selective estrogen receptor modulator; SERD: selective estrogen receptor degrader.

|  | Drug | N_observed_ | N_drug_ | ROR | 95% CI |
| --- | --- | --- | --- | --- | --- |
| AIs | Anastrozole | 223 | 8863 | 1.47 | (**1.28**-1.68) |
|  | Letrozole | 488 | 21172 | 1.34 | (**1.23**-1.47) |
|  | Exemestane | 178 | 9550 | 1.09 | (0.94-1.26) |
| SERM | Tamoxifen | 185 | 9725 | 1.11 | (0.96-1.28) |
| SERD | Fulvestrant | 192 | 10497 | 1.07 | (0.92-1.23) |
| iCDK4/6 | Palbociclib | 697 | 31839 | 1.28 | (**1.19**-1.38) |
|  | Ribociclib | 111 | 7825 | 0.83 | (0.69-1.00) |
|  | Abemaciclib | 40 | 3964 | 0.59 | (0.43-0.80) |

**Supplementary table 5: Disproportionality analysis of neurocognitive impairment with endocrine therapies from the date of the first notification of an adverse event for each drug to the date of the first notification of an adverse event with an inhibitor of CDK4/6**

The relative odds ratio (ROR) and its 95% credibility interval (CI) lower and upper endpoints evaluate the observed-to-expected ratios of neurocognitive impairment (NCI) cases associated with endocrine therapies and inhibitors of CDK4/6 in VigiBase® (from the date of the first notification of an adverse event for each endocrine therapy to January 1^st^, 2014, date of the first notification of an adverse event with an inhibitor of CDK4/6). A lower ROR CI endpoint >1 (**in bold** **type**) denotes an association between a drug and an adverse event. Only one case of NCI was identified with toremifene. A ROR was not calculable and is therefore not presented in the table. AI: aromatase inhibitors; N_drug_: number of adverse events with the drug over the period of interest; N_observed_: number of reports of NCIs with the drug over the period of interest; SERM: selective estrogen receptor modulator; SERD: selective estrogen receptor degrader.

|  | Drug | N_observed_ | N_drug_ | ROR | 95% CI |
| --- | --- | --- | --- | --- | --- |
| AIs | Anastrozole | 510 | 12691 | 1.28 | (**1.17**-1.40) |
|  | Letrozole | 333 | 9596 | 1.10 | (0.99-1.23) |
|  | Exemestane | 159 | 4996 | 1.01 | (0.86-1.18) |
| SERM | Tamoxifen | 564 | 19669 | 0.91 | (0.84-0.99) |
| SERD | Fulvestrant | 91 | 1922 | 1.50 | (**1.22**-1.86) |
